# A Rare Case of Cervical Vagus Nerve Schwannoma in an Adult Patient

**DOI:** 10.7759/cureus.25211

**Published:** 2022-05-22

**Authors:** Andrianos S Tzortzis, Panagiotis Dogantzis, Nikolaos Koliakos, Simeon Tsintzos, George Tzortzis

**Affiliations:** 1 Medicine, National and Kapodistrian University of Athens Medical School, Athens, GRC; 2 Otolaryngology, Panarcadian General Hospital of Tripolis, Tripolis, GRC; 3 3rd Department of Surgery, Attikon University Hospital, Athens, GRC; 4 Oral and Maxillofacial Surgery, Panarcadian General Hospital of Tripolis, Tripolis, GRC

**Keywords:** head and neck neoplasms, case report, surgical treatment, management, vagus nerve schwannoma

## Abstract

Schwannomas of the head and neck are relatively rare benign tumors that derive from the Schwann cells. Schwannomas are usually asymptomatic; however, tumors of bigger size may produce unspecific symptoms due to compression of the adjacent anatomic structures. Vagus nerve schwannomas may present as solitary neck masses, produce hoarseness of voice, or induce paroxysmal cough on palpation, which is also pathognomonic. Preoperative diagnosis is challenging and imaging studies may play a vital role in the diagnosis. Surgical treatment with complete tumor removal is the treatment of choice. In this study, we present a case of vagus nerve schwannoma in an adult male patient.

## Introduction

Schwannomas are slow-growing, benign, encapsulated nerve sheath tumors that derive from Schwann cells [1]. Embryologically, Schwann cells originate from the neural crest cells, and their main role is to support, maintain, and regenerate the neural axons of the peripheral nervous system [2]. Schwannomas can develop from any peripheral nerve with the exception of the olfactory and optic nerves, and they predominantly present in the head and neck region with an incidence of 25-45% [1,3]. They are usually reported in individuals aged between 20 to 50 years and there seems to be no difference in incidence between genders [3]. As the condition is mostly asymptomatic, the role of imaging is of paramount importance in understanding the anatomic relationships with the surrounding tissues and leads to correct diagnosis and proper surgical management. Malignant transformation is rare, but possible, with an incidence rate between 8 and 13.9% [4]. In this report, we discuss a case of a cervical vagus nerve schwannoma in an adult patient.

## Case presentation

A 41-year-old male patient was referred to the outpatient clinic of the Oral and Maxillofacial Surgery Department with a history of a mass in the right lateral neck area. The mass had been present for the last eight years; however, the patient had begun to experience some nonspecific symptoms in the previous year. The main complaints were mild hoarseness of voice, episodes of bradycardia, and syncope. His physical examination revealed a non-tender, soft, mobile, smooth-surfaced mass on the right side of the neck. Notably, the palpation of the neck induced paroxysmal cough. No cervical lymph nodes were palpated.

**Figure 1:**
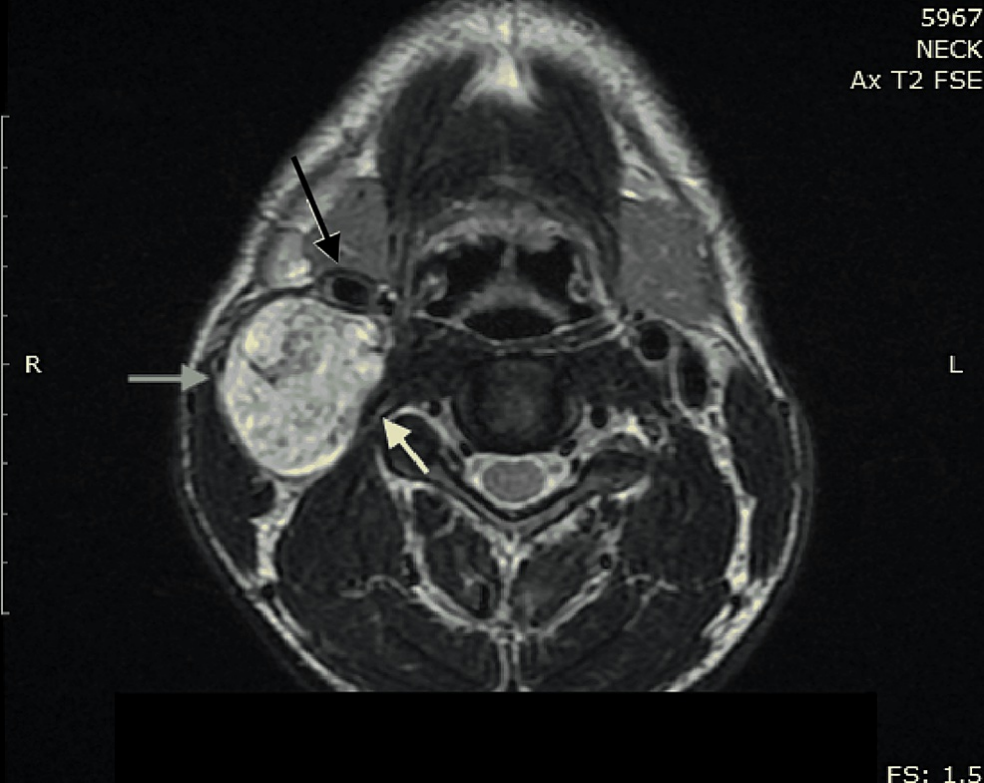
MRI of the neck Axial view. Gray arrow: well-circumscribed, highly-vascularized mass with dimensions of 3.5 x 3.5 x 1.6 cm in the right lateral cervical region directly under the right sternocleidomastoid muscle, separating the internal jugular vein and the carotid artery. Yellow arrow: compressed internal jugular vein. Black arrow: carotid artery MRI: magnetic resonance imaging

The patient underwent an MRI of the neck (Figure 1), which demonstrated a well-circumscribed, highly-vascularized mass with dimensions of 3.5 x 3.5 x 1.6 cm in the right lateral cervical region, directly under the sternocleidomastoid muscle and posteriorly to the common carotid artery and its bifurcation to the external and internal carotid artery. MRI showed separation of the internal jugular vein and the common carotid artery caused by the mass (Figure 1). As a result, schwannoma of the vagus nerve was considered the most possible diagnosis. A preoperative assessment of the motility of the vocal cords was performed, which revealed mild palsy of the right vocal cord.

**Figure 2:**
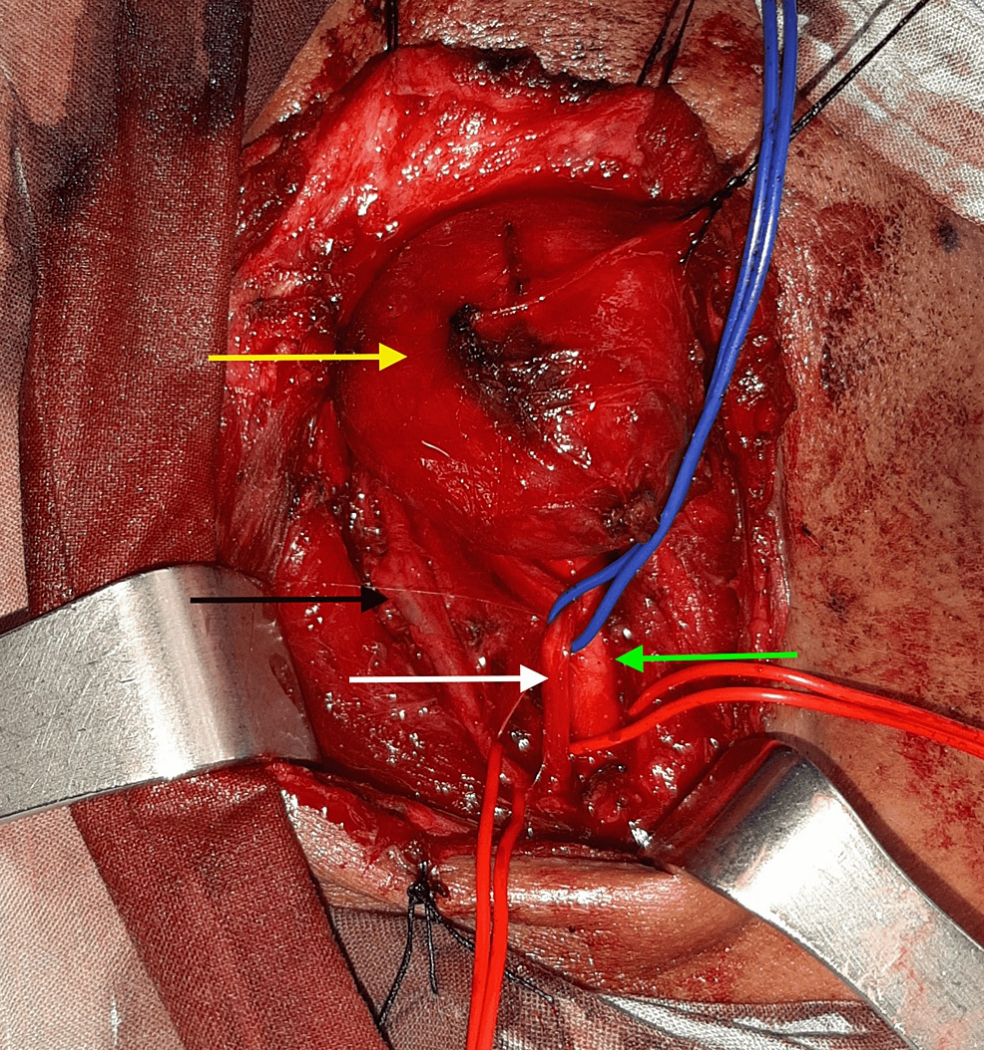
Intraoperative image White arrow: the vagus nerve. Green arrow: carotid artery. Black arrow: internal jugular vein. Yellow arrow: vagus nerve schwannoma

**Figure 3:**
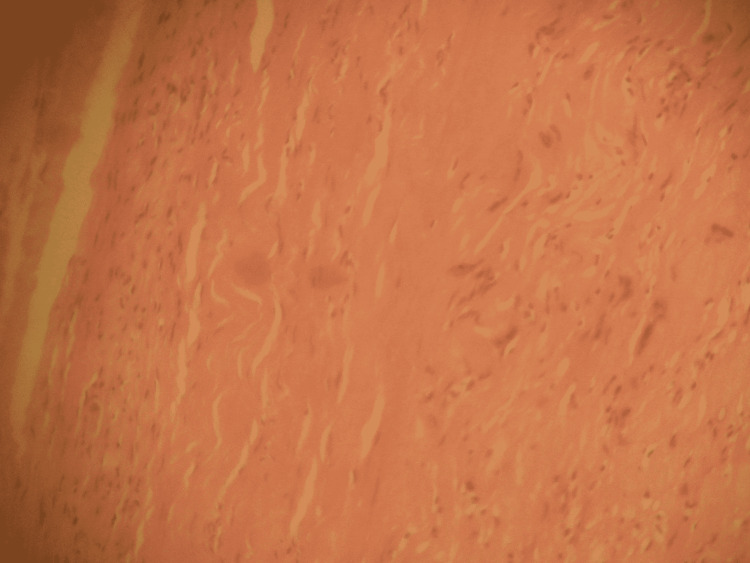
Histological illustration of the mass (H&E x 20)

The patient underwent complete tumor removal under general anesthesia. The incision was placed in an upper neck skin crease extending from the mastoid process toward the hyoid bone, overlying the mass. Then, the skin incision was deepened through the platysma to expose the anterior border of the sternocleidomastoid muscle. The fascia anterior to the sternocleidomastoid muscle was incised and the sternocleidomastoid muscle was retracted laterally to expose the mass. The carotid sheath was dissected and the internal jugular vein, the carotid artery, and the vagus nerve were exposed. The tumor was found to be arising from the vagus nerve and separated the internal jugular vein from the carotid artery. Notably, the internal jugular vein was compressed by the tumor. The entire tumor was safely separated from the nerve and totally excised while preserving the anatomic continuity and the functionality of the nerve (Figure 2). Histological examination confirmed the diagnosis of schwannoma of the vagus nerve (Figure 3).

The postoperative period was uneventful, and the patient was discharged on the fourth postoperative day. Upon follow-up after 12 months, he remains asymptomatic with no signs of recurrence.

## Discussion

Schwannomas of the head and neck are benign tumors with a slow growth rate. However, it is important to note that there is an incidence rate of 8-13.9% of malignant transformation, which should be taken into consideration in the management of schwannoma [4]. They are usually asymptomatic, with the most common presentation being a solitary mass. Other nonspecific symptoms, such as dysphagia, nasal obstruction, and dyspnea in the supine position, occur in the later stages, due to the increased size of the mass compressing the surrounding anatomical structures [4]. In the case of vagus nerve schwannomas, patients occasionally present with hoarseness of voice and a specific pathognomonic symptom of paroxysmal cough upon cervical mass palpation [1,4]. The differential diagnosis of such neck formations should include inflammatory cervical lymphadenitis, metastatic lymphadenopathy, lymphoma, paraganglioma, salivary gland neoplasms, branchial cleft cyst, carotid body tumors, neurofibroma, and carotid aneurysm [5].

Preoperative diagnosis of cervical vagus schwannoma is difficult since the clinical presentation tends to be nonspecific [5]. Preoperative imaging studies and fine-needle aspiration cytology (FNAC), however, can be of value in aiding diagnosis. Moreover, imaging studies also play an important role in surgical planning, as they provide details of the lesion and the surrounding anatomy [1]. In this context, MRI is considered superior to the CT scan, since it provides a more detailed description of the soft tissues [1,4]. Contrast-enhanced CT describes schwannomas with elevated attenuation compared to the surrounding muscle with low to moderate heterogeneous contrast enhancement. Occasionally, internal cystic changes that stem from mucinous degeneration, hemorrhage, or necrosis can be observed. T1-weighted MR images show the isointense signal of the schwannoma compared to the surrounding muscle, while T2-weighted images report a heterogeneously hyperintense signal. Characteristic MR signs of schwannomas include “split fat,” “fascicular,” and “target” [6]. In our case, the MRI sign was fascicular.

Imaging is useful in distinguishing between the entities included in the differential list as it can depict the relationship of the mass with the adjacent structures, and provide some insight into the mass composition. Differentiation between vagal schwannomas and cervical sympathetic chain schwannomas can be made by the displacement of the carotid sheath contents. The latter displace both the carotid artery and internal jugular vein laterally since they are not inside the carotid sheath, while vagal schwannomas tend to separate them. Lymph node involvement can be considered when both the carotid and the jugular vein are displaced medially, as cervical lymph nodes are lateral to the carotid sheath. Carotid body tumors separate the internal and external carotid arteries at the carotid bifurcation level [6]. Ultrasound imaging depicts schwannomas as round or elliptical with clear margins and internal echo reflection. If the originating nerve’s diameter is big enough, ultrasound can depict their connection [1].

The role of FNAC is debatable because schwannomas are deep-seated and usually adjacent to the large vessels, and injury may occur [7]. Hence, FNAC is not recommended, unless malignancy must be ruled out [1,3,7]. In our case, FNAC was avoided because of the close proximity of the mass with the great vessels.

There are two histopathological patterns of schwannomas: Antoni A and B. Both types may coexist. In Antoni A pattern, there is high cellularity, with the cells being arranged in palisades, bundles, or whirls, around central acellular eosinophilic areas, called Verocay bodies [1,4]. Antoni B pattern is characterized by a smaller number of cells along cystic degeneration or xanthomatous changes [1]. In our case, further histological classification was difficult due to heavy degeneration, as a result of the mass existing for so many years.

Complete surgical removal of the tumor is the mainstay of treatment. In the literature, there are two different methods of dealing with schwannomas. One involves the dissection of the nerve fascicle from which the schwannoma originates, removing the schwannoma with its capsule (total resection) with the occasional sacrifice of the vagus nerve, while the other, more conservative, method pertains to the enucleation of the schwannoma from inside its capsule with the preservation of the nerve [1,6].

A recent review by Sandler et al. [6] comparing these two surgical techniques showed that the enucleation method was statistically superior in terms of nerve-sparing and postoperative course. More patients in the enucleation group reported that they were asymptomatic postoperatively. Furthermore, the patients who reported postoperative symptoms of hoarseness or vocal fold paralysis were fewer in the enucleation group compared to those who underwent total excision. However, the postoperative course also depends on the surgeon’s skills and familiarity with the technique as well as the size and location of the lesion. Postoperative side effects of vagus nerve damage also include dysphagia, gastroesophageal reflux disease, early satiety nausea, and heartburn [6]. Recurrence rates of vagal schwannomas are low; however, there is not enough data in the literature to produce statistically substantial results [6]. Partial tumor removal is believed to be associated with higher recurrence rates [1].

Recently, vagus nerve monitoring has been proposed as an additional precautionary measure to ensure fewer incidences of postoperative nerve damage. However, it remains to be determined how useful nerve monitoring is as there have been a few cases that reported nerve monitoring during the procedure [6].

## Conclusions

Vagus nerve schwannomas are rarely occurring neck masses with unusual and atypical symptoms. Imaging is a very helpful modality for diagnosis and surgical planning. It seems that the enucleation method is associated with fewer complications, but this depends on the surgeon’s skills. Recurrence rates are estimated to be very low. In our case, the patient presented with a palpable neck mass and nonspecific symptoms, such as mild hoarseness of voice, episodes of bradycardia, and syncope. Preoperative imaging suggested cervical vagus nerve schwannoma as the most possible diagnosis. Complete tumor removal with dissection and preservation of the vital anatomic structures and the functionality of the vagus nerve under general anesthesia was performed. Upon follow-up after 12 months, the patient remains asymptomatic with no signs of recurrence.
